# Individuals’ Desire for Social Needs Sharing Among Healthcare Providers: Findings from the 2022 Health Information National Trends Survey

**DOI:** 10.1007/s11606-024-09339-9

**Published:** 2025-01-28

**Authors:** Ramona G. Olvera, Christine M. Swoboda, Joshua J. Joseph, Seuli Bose-Brill, Ann Scheck McAlearney, Daniel M. Walker

**Affiliations:** 1 The Center for the Advancement of Team Science, Analytics, and Systems Thinking (CATALYST), College of Medicine, The Ohio State University, Columbus, OH USA; 2 Division of Endocrinology, Diabetes and Metabolism, Department of Internal Medicine, College of Medicine, The Ohio State University, Columbus, OH USA; 3 Division of Internal Medicine, Department of Internal Medicine, College of Medicine, The Ohio State University, Columbus, OH USA; 4 Department of Family and Community Medicine, College of Medicine, The Ohio State University, Columbus, OH USA

**Keywords:** social needs, social determinants of health, trust, data sharing, patient-centered communication

## Abstract

**Background:**

Increasingly, health systems are collecting and using social needs data, yet there is limited information about individuals’ preferences for how social needs information is shared among providers for treatment purposes.

**Objective:**

To explore the connection between experiencing social needs and concerns about healthcare providers sharing social needs information.

**Design and Participants:**

A nationally representative, cross-sectional study of 6252 US community-dwelling adults (≥ 18 years of age) who responded to the Health Information National Trends Survey (HINTS 6) (response rate 28.1%) from March to November 2022.

**Main Measures:**

Core measures include experiencing social needs in the past year (food, transportation, housing issues), and comfort with providers sharing social needs information with each other for treatment purposes. Other key independent variables included patient-centered communication, discrimination, trust, and quality of care.

**Key Results:**

Odds of reporting experiencing a social need varied by age, race, education, and income, yet those 75 years or older (compared to ages 18–34) had higher odds of reporting feeling comfortable with providers sharing information about social needs. Those who reported having experienced discrimination in healthcare had lower odds of reporting comfort with providers sharing information about food (adjusted odds ratio [aOR], 0.63; 95% confidence interval [CI], 0.41-0.98) and transportation (aOR, 0.64; 95%CI, 0.44-0.94) needs. Those who trusted the healthcare system had higher odds of being comfortable with providers sharing information about unmet needs for food (aOR, 1.33; 95%CI, 1.07-1.65). Also, those who report better patient-centered communication and quality of care had higher odds of being comfortable with providers sharing information on unmet social needs.

**Conclusions:**

Individuals’ preferences regarding social needs information sharing for treatment purposes, as well as experience of discrimination, trust in healthcare, quality of care, and patient-provider communication, should be considered in health system efforts to capture social needs information.

**Supplementary Information:**

The online version contains supplementary material available at 10.1007/s11606-024-09339-9.

## BACKGROUND

Healthcare payers and providers are increasingly acknowledging the effect of social factors on patients’ health outcomes.^1^ In response, providers have expanded efforts to understand, collect information about, and refer patients to community resources to attempt to address individual-level non-medical, health-related social needs (social needs).^1–5^ These social needs often include information about food security,^6–8^ housing,^9,10^ transportation,^11^ utilities, job security, education, childcare, and personal safety.^12–14^ Research of practitioners’ perspectives on collecting social needs information from patients has reported that they believe their patients are reluctant to share social needs information.^15,16^ Studies from the patient perspective suggest that patients are generally willing to share information about their social needs,^17–20^ despite some concerns about the timing of data collection during their appointments, privacy and confidentiality, and rapport with the care team member asking questions.^18,21,22^

However, the collection of social needs information is only the first step for healthcare providers to address patients’ social needs.^23^ To develop care plans that address patients’ social needs, providers must then share this information with other medical and non-medical providers that can appropriately resolve identified needs. This next step requires clinicians to have familiarity with social services, either internal or external to their health system, that can support patients^24^ and mechanisms to connect patients to these services, such as sharing a paper list of available resources or including automated electronic health record referrals to agencies or social service providers.^25^ Ideally, the provider/health system-social service connection would involve bi-directional information sharing,^25^ yet little is known about the public’s perspectives about what is done with their social needs information once shared with providers.

The objective of this study is to address this gap by exploring individuals’ perspectives about healthcare providers sharing social needs information with other providers for treatment purposes using national, cross-sectional data from the 2022 iteration of the Health Information National Trends Survey (HINTS). Findings can aid efforts to expand social needs data collection as they can inform providers about areas of individuals’ sensitivity and suggest populations that may require thoughtful communication regarding how this data may be used and shared across treatment services.

## METHODS

### Study Sample

HINTS was created by the National Cancer Institute and asks questions about cancer beliefs, health behaviors, health information use, healthcare beliefs and use, and more.^26^ HINTS has been fielded 16 times between 2003 and 2022 in the United States of America (US) and is administered to random samples of non-institutionalized adults aged 18 or older residing in the US.^27^ All HINTS administrations are de-identified before being made available, have been approved by the Westat Institutional Review Board, and have been deemed exempt from approval or consent for subsequent analyses.^28^ The HINTS survey is sent with a $2 incentive to encourage participant completion.^27^

HINTS 6 was administered from March to November 2022 yielding 6252 responses for a response rate of 28.1%.^27^ Data were collected in English and Spanish by mail and online. Mailed surveys were collected in two stages: first from randomly selected residential addresses from the US Postal Service, and second from the selected adult in the household with the nearest upcoming birthday.^27^

In 2022, HINTS added a social determinants of health (SDOH) module with seven questions; only data from the 2022 administration of HINTS were analyzed because of the addition of new questions. Data were analyzed using survey weighting to make the results more generalizable to the US population. Survey weights were designed based on population estimates from the American Community Survey and jackknife replicate weights were used to provide bias-corrected variance estimates.^29^

### Independent and Dependent Variables

The social needs risk factor dependent variables from the SDOH module included: “In the past 12 months how often was the following true” with the options: “Someone in your household cut the size of meals or skipped meals because there wasn’t enough money for food?”, “Someone in your household was not able to afford to eat balanced meals?”, “Lack of reliable transportation kept someone in your household from medical appointments, work, or from getting things needed for daily living?”, and “Someone in your household was worried about being forced to move (for example, because of eviction or foreclosure)?”^27^ Answers were dichotomized into “Often/Sometimes” and “Never” for analysis. The dependent variables of interest in this study around comfort with providers sharing social needs information with other providers for treatment purposes were assessed using the hypothetical questions: “If you were experiencing issues with affording or accessing healthy food,” “If you were experiencing issues with transportation that make it difficult getting to work or medical appointments,” and “If you were experiencing issues with housing,” followed by “how comfortable would you be with your health care providers sharing that information with each other for treatment purposes?” with the responses turned into a binary variable for analysis: “Very/somewhat comfortable” and “very/somewhat uncomfortable.”^27^

Independent variables including trust in healthcare, past discrimination during medical care, and quality of care were assessed.^27^ A validated patient-centered communication (PCC) scale included seven questions regarding perceptions of health providers’ communication skills.^30^ The PCC questions used a Likert-type 4-point scale: Always (1), Usually (2), Sometimes (3), and Never (4), and for this analysis, scale scores were created by reverse-scoring all items and summing them, so that the minimum score, 7, would be all “Never” and the maximum score, 28, would be “Always.” Sociodemographic factors were selected as covariates to include in our analyses based on prior work identifying correlations between them and the experience of discrimination and/or social needs: gender (male/female), race and ethnicity (non-Hispanic White/non-Hispanic Black/Hispanic/non-Hispanic Other (including Asian, American Indian or Alaska Native, Native Hawaiian or Other Pacific Islander, and multiple races mentioned)), age (18–34/35–49/50–64/66–74/75+), education (high school diploma or less/some college/college graduate or more), income (less than $35,000/$35,000–$75,000/more than $75,000), health insurance (insured/uninsured), and marital status (single/married or living as married/separated or divorced/widowed).^31^ Extended details of these measures are included in Appendix 1.

### Statistical Approach

Weighted descriptive statistics for each variable were analyzed. Chi-square tests were performed to assess significant differences between demographic variables and social needs risk factors. Multivariable logistic regression models were used to explore associations between the dependent variables and independent and sociodemographic variables. All models were adjusted to control for sociodemographic variables (i.e., gender, race and ethnicity, age, education, income). We used complete case analysis to account for missing values operating under the assumption that data is missing at random and because missingness was less than 10% for dependent variables of interest.^32^ Results were considered significant for *p*-values less than 0.05. All analyses were conducted using Stata version 17 (2021, StataCorp LLC, College Station, TX).

## RESULTS

Table 1 shows weighted sample demographic characteristics for each social need. The majority of the sample was female (50.8%), non-Hispanic White (61.2%), insured (89.3%), and married/living as married (55.9%). There were significantly different proportions that responded “Often” or “Sometimes” to any of the social needs questions versus “Never” by age, race and ethnicity, education, and income (*p* < 0.05).

**Table 1:** Sample Demographic Characteristics for Each Social Need (Skip Meals, Afford Meals, Transportation, Worry About Being Forced to Move)

	Total *n* (weighted %)	Often/sometimes skip meals *n* (weighted %)	*p*-value*	Often/sometimes can’t afford meals *n* (weighted %)	*p*-value*	Often/sometimes lack transportation *n* (weighted %)	*p*-value*	Often/sometimes worry about being forced to move *n* (weighted %)	*p*-value*
	*N* = 6252	*n* = 793 (12.6%)		*n* = 912 (14.6%)		*n* = 696 (11.1%)		*n* = 597 (9.5%)	
Gender			0.39		0.17		0.14		0.09
Male	2307 (49.2)	243 (14.6)		272 (16.6)		211 (11.6)		190 (9.4)	
Female	3535 (50.8)	519 (16.0)		605 (19.0)		453 (13.9)		379 (11.9)	
Age			<0.001		<0.001		<0.001		<0.001
18–34	939 (25.9)	202 (22.9)		228 (27.3)		156 (20.6)		143 (15.9)	
35–49	1240 (25.3)	194 (17.9)		229 (20.8)		167 (13.4)		145 (13.9)	
50–64	1772 (27.3)	235 (13.3)		269 (15.3)		209 (10.8)		203 (9.6)	
65–74	1356 (13.0)	114 (8.1)		134 (9.9)		91 (6.9)		66 (3.9)	
75 or older	847 (8.6)	39 (6.1)		43 (6.2)		64 (7.5)		32 (3.7)	
Race and ethnicity			0.001		0.001		0.001		<0.001
Non-Hispanic White	3203 (61.2)	305 (11.9)		353 (13.7)		241 (8.9)		238 (7.9)	
Non-Hispanic Black	889 (11.0)	150 (22.9)		166 (24.0)		156 (15.6)		117 (14.5)	
Hispanic	1001 (17.1)	193 (18.2)		228 (24.2)		155 (15.5)		120 (12.4)	
Non-Hispanic Other	472 (10.8)	78 (23.5)		93 (26.8)		76 (7.6)		70 (20.6)	
Education			<0.001		<0.001		<0.001		<0.001
High school graduate and below	1455 (28.5)	256 (19.1)		273 (20.8)		234 (16.3)		155 (10.0)	
Some college	1672 (38.9)	288 (18.9)		349 (23.2)		247 (16.2)		224 (14.8)	
College graduate or more	2721 (32.6)	221 (8.0)		259 (9.4)		186 (6.0)		191 (6.5)	
Income			<0.001		<0.001		<0.001		<0.001
Less than $35,000	1688 (25.5)	417 (31.9)		474 (36.0)		386 (28.6)		294 (19.4)	
$35,000–$75,000	1669 (30.1)	250 (19.3)		290 (22.4)		183 (14.5)		182 (14.4)	
Greater than $75,000	2163 (44.4)	81 (4.1)		100 (5.5)		77 (3.5)		84 (4.1)	
Health insurance			0.001		<0.001		0.004		0.005
Uninsured	521 (10.7)	128 (25.9)		141 (32.6)		102 (23.5)		86 (17.4)	
Insured	5605 (89.3)	658 (14.2)		765 (16.4)		589 (11.8)		505 (10.1)	
Marital status			<0.001		<0.001		<0.001		<0.001
Single	1119 (31.2)	234 (24.5)		263 (27.2)		207 (21.8)		164 (16.7)	
Married/living as married	2997 (55.9)	291 (10.5)		337 (12.7)		224 (7.3)		200 (6.7)	
Separated/divorced	1075 (8.1)	184 (17.8)		213 (22.3)		176 (18.3)		159 (15.4)	
Widowed	646 (4.8)	52 (9.1)		64 (12.6)		59 (11.8)		43 (9.6)	

^*^Significance calculated with comparison to responses of “Never” for each social need (skip meals, afford meals, transportation, worry about being forced to move)

Table 2 shows the adjusted association of demographic characteristics for each social needs. The logistic regression models showed that non-Hispanic Other race respondents had higher odds of experiencing any of the four social needs (aOR (95%CI): skipping meals, 1.96 (1.10–3.48); unable to afford meals, 1.83 (1.02–3.28); lacking transportation, 2.62 (1.27–5.40); worried about being forced to move, 2.40 (1.24–4.62). Additionally, those in the age groups 65–74 and 75+ and those with incomes $75,000 or higher had lower odds of experiencing any social needs.

**Table 2:** Association of Demographic Characteristics for Each Social Needs

	Often/sometimes skip meals Adjusted OR (95%CI)*	Often/sometimes can’t afford meals Adjusted OR (95%CI)*	Often/sometimes lack transportation Adjusted OR (95%CI)*	Often/sometimes worried about being forced to move Adjusted OR (95%CI)*
Gender				
Male	Ref	Ref	Ref	Ref
Female	1.08 (0.81–1.44)	1.18 (0.92–1.51)	1.12 (0.84–1.49)	1.22 (0.87–1.70)
Age				
18–34	Ref	Ref	Ref	Ref
35–49	0.97 (0.64–1.47)	0.85 (0.55–1.32)	0.80 (0.49–1.32)	1.19 (0.81–1.74)
50–64	**0.64 (0.41–0.99)**	**0.52 (0.34–0.78)**	0.64 (0.39–1.04)	0.75 (0.48–1.15)
65–74	**0.28 (0.16–0.49)**	**0.25 (0.15–0.42)**	**0.29 (0.16–0.53)**	**0.17 (0.09–0.35)**
75 or older	**0.19 (0.07–0.57)**	**0.12 (0.04–0.36)**	**0.29 (0.12–0.68)**	**0.11 (0.05–0.27)**
Race and ethnicity				
Non-Hispanic White	Ref	Ref	Ref	Ref
Non-Hispanic Black	1.04 (0.68–1.59)	0.93 (0.63–1.37)	1.33 (0.87–2.04)	1.05 (0.68–1.60)
Hispanic	0.85 (0.60–1.23)	0.98 (0.65–1.47)	1.32 (0.77–2.24)	0.99 (0.58–1.71)
Non-Hispanic Other	**1.96 (1.10–3.48)**	**1.83 (1.02–3.28)**	**2.62 (1.27–5.40)**	**2.40 (1.24–4.62)**
Education				
High school graduate and below	Ref	Ref	Ref	Ref
Some college	**1.48 (1.02–2.14)**	**1.83 (1.24–2.72)**	1.29 (0.87–1.90)	**1.06 (1.27–3.02)**
College graduate or more	0.76 (0.46–1.24)	0.84 (0.53–1.34)	0.64 (0.39–1.04)	1.04 (0.66–1.64)
Income				
Less than $35,000	Ref	Ref	Ref	Ref
$35,000–$75,000	**0.49 (0.32–0.75)**	**0.50 (0.34–0.74)**	**0.45 (0.30–0.68)**	0.71 (0.44–1.14)
Greater than $75,000	**0.09 (0.05–0.14)**	**0.10 (0.06–0.17)**	**0.11 (0.06–0.20)**	**0.17 (0.10–0.27)**
Health insurance				
Uninsured	Ref	Ref	Ref	Ref
Insured	0.81 (0.50–1.33)	0.71 (0.45–1.10)	0.79 (0.47–1.34)	0.92 (0.53–1.60)
Marital status				
Single	Ref	Ref	Ref	Ref
Married/living as married	0.81 (0.58–1.13)	0.89 (0.64–1.25)	**0.62 (0.46–0.85)**	0.71 (0.50–1.01)
Separated/divorced	0.90 (0.62–1.29)	1.19 (0.84–1.68)	1.15 (0.81–1.64)	1.33 (0.88–2.00)
Widowed	0.52 (0.25–1.11)	0.93 (0.48–1.78)	0.89 (0.45–1.77)	1.38 (0.70–2.74)

All estimates adjusted for demographic characteristics gender, race and ethnicity, age, education, and income

^*^Each model compares responses of “Often/Sometimes” with responses of “Never”

Table 3 shows the association of demographics and comfort with providers sharing social needs information with other providers (see Appendix 2 for percentages of respondents comfortable sharing social needs information with other providers). Adults aged 75 or older had the highest odds of feeling comfortable with their healthcare providers sharing information about food (aOR (95%CI), 1.62 (1.06–2.46)) or transportation (aOR (95%CI), 1.50 (1.01–2.24)) issues.

**Table 3:** Association of Demographics and Comfort Providers Sharing Social Needs Information with Other Providers

	Comfortable with providers sharing information about food access issues Adjusted OR (95%CI)*	Comfortable with providers sharing information about transportation issues Adjusted OR (95%CI)*	Comfortable with providers sharing information about housing issues Adjusted OR (95%CI)*
Gender			
Male	Ref	Ref	Ref
Female	1.05 (0.87–1.27)	1.09 (0.91–1.31)	1.01 (0.84–1.23)
Age			
18–34	Ref	Ref	Ref
35–49	0.96 (0.67–1.37)	1.11 (0.76–1.63)	1.03 (0.72–1.46)
50–64	0.90 (0.683–1.20)	0.91 (0.66–1.25)	0.89 (0.66–1.21)
65–74	1.05 (0.75–1.47)	1.10 (0.75–1.62)	1.04 (0.73–1.47)
75 or older	**1.62 (1.06–2.46)**	**1.50 (1.01–2.24)**	1.46 (0.99–2.16)
Race and ethnicity			
Non-Hispanic White	Ref	Ref	Ref
Non-Hispanic Black	1.09 (0.83–1.44)	1.31 (0.97–1.75)	1.06 (0.82–1.38)
Hispanic	1.09 (0.78–1.52)	1.16 (0.83–1.62)	1.06 (0.77–1.48)
Non-Hispanic Other	1.02 (0.71–1.47)	0.88 (0.63–1.22)	0.97 (0.68–1.39)
Education			
High school graduate and below	Ref	Ref	Ref
Some college	1.01 (0.74–1.36)	1.03 (0.77–1.38)	1.03 (0.78–1.36)
College graduate or more	1.17 (0.85–1.61)	1.22 (0.91–1.62)	1.18 (0.88–1.60)
Income			
Less than $35,000	Ref	Ref	Ref
$35,000–$75,000	0.92 (0.66–1.28)	0.95 (0.68–1.34)	0.92 (0.66–1.28)
Greater than $75,000	0.97 (0.66–1.43)	0.91 (0.64–1.29)	0.83 (0.59–1.18)
Health insurance			
Uninsured	Ref	Ref	Ref
Insured	0.99 (0.67–1.47)	1.04 (0.69–1.58)	1.00 (0.72–1.38)
Marital status			
Single	Ref	Ref	Ref
Married/living as married	0.90 (0.66–1.21)	1.03 (0.74–1.43)	1.11 (0.81–1.52)
Separated/divorced	1.03 (0.73–1.47)	1.25 (0.85–1.83)	1.21 (0.82–1.79)
Widowed	0.81 (0.55–1.22)	0.89 (0.55–1.42)	0.95 (0.64–1.40)

All estimates adjusted for demographic characteristics gender, race and ethnicity, age, education, and income

^*^Each model compares responses of somewhat/very comfortable to responses of somewhat/very uncomfortable with providers sharing information about social needs with other providers

Table 4 shows the relationship between comfort with providers sharing information about food, transportation, and housing issues with other providers for treatment purposes by PCC score, discrimination, trust the healthcare system, and quality of care. Controlling for demographic variables, for every 1 point increase in PCC score, there were higher odds of being comfortable with providers sharing information about food (aOR (95%CI), 1.07 (1.04–1.09)), transportation (aOR (95%CI), 1.07 (1.54–1.10)), or housing issues (aOR (95%CI), 1.07 (1.04–1.09)). Those who had experienced discrimination had lower odds of being comfortable with providers sharing food or transportation information. Those who reported “Very good” or “Excellent” quality of care had higher odds of being comfortable with providers sharing all three social needs.

**Table 4:** Model Results of Comfort with Providers Sharing Information About Social Needs with Other Providers by Patient-Centered Communication (PCC), Discrimination, Trust, and Quality of Healthcare

	Comfortable with providers sharing information about food access issues* Adjusted OR (95%CI)	Comfortable with providers sharing information about transportation issues* Adjusted OR (95%CI)	Comfortable with providers sharing information about housing issues* Adjusted OR (95%CI)
PCC score (7–28)†	**1.07 (1.04–1.09)**	**1.07 (1.05–1.10)**	**1.07 (1.04–1.09)**
Ever discriminated against			
No	Ref	Ref	Ref
Yes	**0.63 (0.41–0.98)**	**0.64 (0.44–0.94)**	0.68 (0.46–1.02)
Trust the healthcare system			
Somewhat/a little/not at all	Ref	Ref	Ref
Very	**1.33 (1.07–1.65)**	1.24 (1.00–1.55)	**1.22 (1.01–1.48)**
Quality of care in past 12 months			
Fair/poor	Ref	Ref	Ref
Good	1.05 (0.68–1.64)	**1.67 (1.08–2.60)**	1.37 (0.93–2.02)
Very good	**1.63 (1.08–2.46)**	**2.26 (1.57–3.24)**	**1.74 (1.28–2.37)**
Excellent	**2.13 (1.44–3.15)**	**3.16 (2.16–4.62)**	**2.50 (1.76–3.57)**

All estimates adjusted for demographic characteristics gender, race and ethnicity, age, education, and income

^*^Each model compares responses of somewhat/very comfortable to responses of somewhat/very uncomfortable with providers sharing information about social needs with other providers

^†^The aORs presented here are for every 1-point increase in PCC score

## DISCUSSION

The collection and sharing of individual social needs data in efforts to address the effects of unmet social needs is of increasing interest to healthcare providers, yet there is limited research on perspectives about the use of individuals’ data once collected by providers. We aimed to address this knowledge gap by exploring the factors influencing individuals’ comfort having their food, transportation, and housing needs shared among providers for the purpose of their treatment. We found that those from younger, lower income, non-Hispanic Other race and ethnic group, and/or with some college had higher odds of reporting social needs including food insecurity, transportation challenges, and housing instability. In terms of comfort sharing social needs, the majority of respondents were comfortable with providers sharing their social needs information and there was no significant difference across gender, race and ethnicity, education, nor income. Only those 75 years or older were more likely to report being comfortable with providers sharing their social needs data with each other for treatment. Notably, adjusting for demographic variables, those who reported experiencing very good or excellent care and/or experiencing better communication with providers had higher odds of reporting feeling comfortable with providers sharing information about their social needs. Those who experienced discrimination, however, had lower odds of reporting feeling comfortable having information about their food or transportation issues shared. Finally, those who reported being very trusting of the healthcare system had higher odds of reporting being comfortable sharing information about their food-related social needs.

Our findings about age, educational, and economic differences associated with experiences of social needs parallel results from other research, although study results have varied.^33^ However, our finding about the absence of differences between racial and ethnic groups is an exception to past research, perhaps because of national representation of this study’s sample compared to those using single-location data. For instance, in a study of a non-safety net population, those who identified as Black or Latino and those who were younger faced more social risks compared to White and older respondents.^34^ Data from another study of a single health system found that Hispanic patients (compared to Black and White patients) and those between 30 and 60 years of age had the greatest individual social risks, including housing and food insecurity.^35^ Our results seem unique in that only the non-Hispanic Other group had higher odds of having food and transportation insecurities compared to non-Hispanic Whites. Regarding differences in social needs based on educational obtainment, those who have completed some college but not graduated may have included additional expenses for their education but not have complete a degree to fully realize this investment.^36^

Our findings also highlight that distinctions among those who experience social needs do not translate to differences in who is willing to have information about those social needs shared among healthcare providers to inform medical treatment. This finding may add insight to results from prior studies reporting the disconnect between those experiencing social needs and those seeking assistance through the healthcare system to address those needs.^5,20,37^ This discrepancy was likewise observed in our data: while most people were comfortable with providers sharing their social needs, the only group who had significantly higher odds of being comfortable with providers sharing data about food, transportation, and housing issues was those aged 75 or older and this older age group also had lower odds of reporting experiencing social needs.

The analysis of the three areas of social needs in this study — food insecurity, transportation, and housing — reflects the social needs information most commonly assessed and/or addressed in healthcare settings after intimate partner violence.^38,39^ However, few institutions are collecting all needs information;^38^ thus, we have opted to report these three social needs areas separately. While other studies focusing on patients’ preferences in sharing social needs information and obtaining resources report systemic and individual issues of stigma in addition to other barriers,^40,41^ we are unaware of any research on the differences in preferences in sharing information about food insecurity, housing, and transportation needs. We imagine that individuals may have similar concerns about stigma and other barriers related to providers sharing their social needs information with other providers as they may have about sharing information with providers initially.

As De Marchis and colleagues posit, interest in receiving assistance to address social needs from medical providers may be mediated by other factors such as past health system discrimination and trust in providers.^42^ Our findings support these suggestions, and additionally clarify the important role of PCC in willingness to have social needs information shared for treatment purposes. PCC may help draw the link between collecting the social needs information and resolving the unmet need — a critical piece in building trust between healthcare providers and their patients. PCC may require additional training, yet the costs and benefits of this would need to be evaluated in order to solidify this approach.^43^

Healthcare providers’ efforts to screen patients’ social needs are increasing, yet our findings suggest that caution is warranted in the handling of this information. Health systems and providers collecting data on social needs should recognize that a one-size-fits-all approach may not serve the needs of all equally.^25^ For instance, if a healthcare provider’s goal is to aid those with identified social needs, a relationship-centered approach^44^ may help address the potential discomfort of younger patients or those from lower socioeconomic backgrounds. For example, there is a need for additional qualitative exploration of differences among population groups in perceived barriers to and facilitators of the sharing of social needs information within healthcare settings. Further, additional mixed methods research may be explored to evaluate different strategies aimed at improving acceptance of social needs screening strategies.

Alternatively, experts have cautioned that integration of social needs information into care may have unintended consequences and may even impede efforts to improve health equity,^45,46^ particularly for those who have experienced past discrimination within or lack of trust in the health system. Some researchers have suggested that resources, such as lists of agencies or website-based resource finders (e.g., UniteUs, findhelp.org), should be provided to all patients regardless of their response to social needs questions.^47,48^ Approaches to collect information about social needs may require detailed clarifications that inform patients on how and with whom this data may be used which will allow patients to control social needs information in their medical records. Particularly for those with social needs, further research is needed to gather insight from diverse individuals into the best ways to ensure that the patient-centered and health equity aspects of both the collection and use of social needs data are considered and addressed.

### Limitations

Our study has some limitations. Large national surveys, such as HINTS, face non-response bias, especially from those from lower socioeconomic households.^28,49^ Also, HINTS is only offered in English and Spanish, potentially limiting responses from households with other language preferences. While these limitations may underestimate results of those with social needs, HINTS attempts to mitigate this selection bias by providing standard weighting procedures which we utilized. Compared with other research on social needs,^33^ our findings fall within the expected range of social needs prevalence. However, the use of the jackknife replication used to estimate survey weights is incompatible with imputation, prohibiting our use of multiple imputation to account for missing data and requiring a complete case analysis. This choice preferences generalizability relative to internal validity, yet is balanced by the low percent (<10%) of missing data for the dependent variables and relatively large remaining sample sizes. Additionally, the statistical associations were interpreted without correction for multiple comparisons. Typical multiple-comparison corrections assume that tests are independent and are too conservative for correlated hypotheses as we have here. A further limitation is the lack of questions in HINTS about other chronic diseases, and thus we are unable to analyze the results by comorbidities. A final limitation is that the novel SDOH section in HINTS asks respondents about a complex topic using seven questions. The questions did not assess patients’ perspectives on sharing information across sectors, particularly with community-based organizations, a key component to address unmet social needs. Thus, we only can report on how respondents noted feeling about healthcare providers sharing social needs information with other healthcare providers, rather than fully explore how respondents might feel about sharing information about these needs with healthcare providers in the first place, or if they might seek help from community resources that healthcare providers may offer.

## CONCLUSION

Both collecting information about social needs and connecting people with assistance to address their social needs are challenging and require great care. The findings from this research suggest individual factors, such as age and past experiences within the healthcare system impact individuals’ comfort in having their social needs information shared among providers for treatment purposes. Even as most people are comfortable having providers share social needs information among other providers, our findings offer that those who experience social needs may be the least comfortable with their needs being shared. Institutional processes, and broader policies, concerning how social information is gathered and used should be guided by robust engagement of diverse stakeholders to ensure patient autonomy and minimize harm, especially discrimination. Supporting providers in building trust and increasing PCC may help maximize patient willingness to communicate sensitive information about social needs, thus helping promote collaboration needed for SDOH-informed clinical care.

## Supplementary Information

Below is the link to the electronic supplementary material.

Supplementary file1 (DOCX 21 KB)

Supplementary file2 (DOCX 98 KB)
