# Supplementary material for: Individuals’ Desire for Social Needs Sharing Among Healthcare Providers: Findings from the 2022 Health Information National Trends Survey
Source: J Gen Intern Med. 2025 Jan 28;41(2):374–82. doi: 10.1007/s11606-024-09339-9 (PMC12894597; doi:10.1007/s11606-024-09339-9)
Supplement: Supplementary file 1 — Supplementary file1 (DOCX 21 KB) [file 11606_2024_9339_MOESM1_ESM.docx]

Appendix 1: Independent and Dependent Variables

The social needs risk factor dependent variables from the SDOH module used included: “In the past 12 months how often was the following true - Someone in your household cut the size of meals or skipped meals because there wasn’t enough money for food?”, “In the past 12 months how often was the following true - Someone in your household was not able to afford to eat balanced meals?”, “ In the past 12 months how often was the following true - Lack of reliable transportation kept someone in your household from medical appointments, work, or from getting things needed for daily living?“, and “In the past 12 months how often was the following true - Someone in your household was worried about being forced to move (for example, because of eviction or foreclosure)?” with the options: “Often,” “Sometimes,” and “Never”.^1^ These were dichotomized into “Often/Sometimes” and “Never” for analysis.

The dependent variables of interest in this study around comfort sharing social needs information with other providers for treatment purposes were assessed using the questions: “If you were experiencing issues with affording or accessing healthy food, how comfortable would you be with your health care providers sharing that information with each other for treatment purposes?”, “If you were experiencing issues with transportation that make it difficult getting to work or medical appointments, how comfortable would you be with your health care providers sharing that information with each other for treatment purposes?”, and “If you were experiencing issues with housing, how comfortable would you be with your health care providers sharing that information with each other for treatment purposes?“ with the responses “Very comfortable,” “Somewhat comfortable,” “Somewhat uncomfortable,” and “Very uncomfortable” turned into a binary variable for analysis: “Very/somewhat comfortable” and “very/somewhat uncomfortable”.^1^

Independent variables assessing healthcare communication and quality were also used. Trust in healthcare was assessed with “How much do you trust the health care system (for example, hospitals, pharmacies, and other organizations involved in health care)?” with the options “Very,” “Somewhat,” “A little,” and “Not at all” aggregated into “Very” versus “Somewhat/A little/Not at all” for analysis.^1^ Discrimination was assessed with: “Have you ever been treated unfairly or been discriminated against when getting medical care because of your race or ethnicity?” with the options of “Yes” or “No”.^1^ For quality of care, the survey asked “Overall, how would you rate the quality of health care you received in the past 12 months” with the options “Excellent”, “Very Good”, “Good”, “Fair”, and “Poor”, and for analysis “Fair” and “Poor” were combined.^1^ A scale assessing patient-centered communication (PCC) included 7 questions with the wording: “In the past 12 months how often did your doctors, nurses or other health care professionals…”: 1) “Give you the chance to ask all the health-related questions you had”; 2) “Give the attention you needed to your feelings and emotions”; 3) “Involve you in decisions about your health care as much as you wanted”; 4) “Make sure you understood the things you needed to do to take care of your health”; 5) “Explain things in a way you could understand”; 6) “Spend enough time with you”; and 7) “Help you deal with feelings of uncertainty about your health or health care”.^2^ The PCC questions used a Likert-type four-point scale: Always (1), Usually (2), Sometimes (3) and Never (4), and for this analysis, scale scores were created by reverse-scoring all items and summing them, so that the minimum score, 7, would be all “Never” and the maximum score, 28, would be “Always”. The quality of care and PCC questions were only answered by respondents who had seen a provider at least once in the past year.

Sociodemographic factors that may affect discrimination or SDOH were used as covariates in our analysis: gender (male/female), race and ethnicity (non-Hispanic White/non-Hispanic Black/Hispanic/non-Hispanic Other (including Asian, American Indian or Alaska Native, Native Hawaiian or Other Pacific Islander, and multiple races mentioned)), age (18-34/35-49/50-64/66-74/75+), education (high school diploma or less/some college/college graduate or more), income (less than $35,000/$35,000-$75,000/more than $75,000), health insurance (insured/uninsured), and marital status (single/married or living as married/separated or divorced/widowed).^3^

**Reference**

1. Westat. Health Information National Trends Survey 6 (HINTS 6) Methodology Report [Internet]. Bethesda, MD: National Cancer Institute; 2023 [cited 2023 Nov 21]. Available from: https://hints.cancer.gov/docs/methodologyreports/HINTS_6_MethodologyReport.pdf

2. Moser RP, Trivedi N, Murray A, Jensen RE, Willis G, Blake KD. Patient-Centered Communication (PCC) scale: Psychometric analysis and validation of a health survey measure. PLOS ONE 2022;17(12):e0279725.

3. Davis BA. Discrimination: A Social Determinant Of Health Inequities. Health Aff Blog Health Aff Forefr [Internet] 2020 [cited 2024 Oct 3];Available from: https://www.healthaffairs.org/content/forefront/discrimination-social-determinant-health-inequities
