# Supplementary material for: Individuals’ Desire for Social Needs Sharing Among Healthcare Providers: Findings from the 2022 Health Information National Trends Survey
Source: J Gen Intern Med. 2025 Jan 28;41(2):374–82. doi: 10.1007/s11606-024-09339-9 (PMC12894597; doi:10.1007/s11606-024-09339-9)

Appendix 2: Weighted and unadjusted percentage of respondents comfortable with healthcare providers sharing different social needs with other providers.

Unadjusted but weighted estimates show that more than half of respondents were comfortable with healthcare providers sharing their social needs information with other providers, with the highest percentage very or somewhat comfortable with sharing transportation issues (61.9%) followed by food access (58.9%) and housing issues (56.7%).


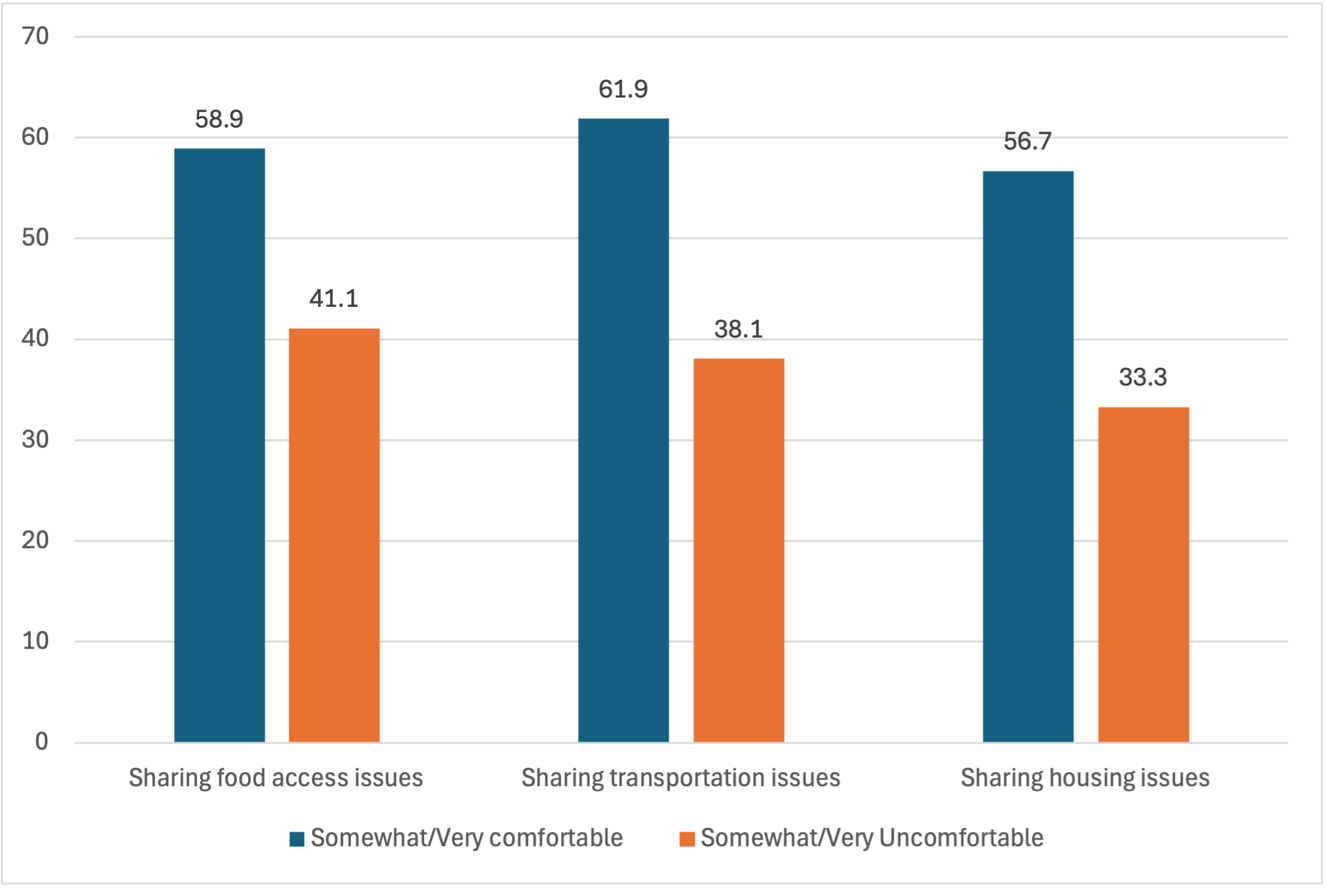

Supplement: Supplementary file 2 — Supplementary file2 (DOCX 98 KB) [file 11606_2024_9339_MOESM2_ESM.docx]
